# Effect of Polyphenol-Rich Diet Combined with Leucine, Vitamin D3, and Magnesium Supplementation on Self-Reported Mobility and Health Perception in Adults at Risk of Sarcopenia: A 3-Months Quasi-Experimental Study

**DOI:** 10.3390/life16040554

**Published:** 2026-03-27

**Authors:** Diana-Lidia Tache-Codreanu, Ana-Maria Tache-Codreanu, Georgeta Stefan, Magdalena Rodica Trăistaru, Elena Rusu, Andrei Tache-Codreanu, Corina Sporea

**Affiliations:** 1 Medical Rehabilitation Department, Colentina Clinical Hospital, 020125 Bucharest, Romania; dianatache@yahoo.com; 2 Department of Medical, Surgical and Preventive Disciplines, Faculty of Medicine, Titu Maiorescu University, 031593 Bucharest, Romania; 3 Faculty of Medicine and Farmacy, University of Medicine and Pharmacy “Carol Davila”, 020021 Bucharest, Romania; ana-maria.tache2024@stud.umfcd.ro; 4 Faculty of Veterinary Medicine, University of Agronomic Sciences and Veterinary Medicine, 050097 Bucharest, Romania; 5 Department of Physiotherapy, University of Medicine and Pharmacy of Craiova, 200349 Craiova-Dolj, Romania; rodicatraistru@hotmail.com; 6 Department of Preclinical Disciplines, Faculty of Medicine, Titu Maiorescu University, 031593 Bucharest, Romania; elenarusu98@yahoo.com; 7 Faculty of Theatre, National University of Theatre and Film “I.L. Caragiale”, 021452 Bucharest, Romania; 8 Faculty of Midwifery and Nursing, University of Medicine and Pharmacy “Carol Davila”, 020021 Bucharest, Romania; corina.sporea@gmail.com; 9 National Teaching Center for Children’s Neurorehabilitation “Robanescu-Padure”, 041408 Bucharest, Romania

**Keywords:** sarcopenia, polyphenol, gut microbiota, muscle strength, nutritional supplementation, leucine, vitamin D, rehabilitation, functional performance

## Abstract

Background: Sarcopenia is characterized by progressive muscle weakness, impaired physical function, and reduced quality of life. Multimodal strategies combining rehabilitation and nutritional support that influence gut microbiota may help improve functional outcomes in adults at risk of sarcopenia. Objective: To evaluate whether a polyphenol-rich dietary recommendation associated with a nutritional supplement containing leucine, vitamin D3, and magnesium (SarcoDYN^®^), in the context of a standardized rehabilitation program, provides superior improvements in functional and patient-reported outcomes compared with rehabilitation alone. Methods: This quasi-experimental, non-randomized retrospective study included 28 adults at risk of sarcopenia, divided into a study group (rehabilitation + polyphenol-rich diet + SarcoDYN^®^) and a control group (rehabilitation only). Assessments were performed at baseline and after 3 months. Outcomes included SARC-F score, handgrip strength (dominant and non-dominant), sit-to-stand performance, perceived mobility, and perceived health status. Within- and between-group comparisons were conducted using appropriate parametric or non-parametric tests, and Spearman correlation analysis explored associations between functional, strength, and perceptual variables. Results: Both groups demonstrated significant within-group improvements in functional and patient-reported outcomes. At follow-up, the study group showed significantly better subjective outcomes, including lower SARC-F scores (U = 30.0, *p* = 0.002), higher perceived mobility (U = 40.0, *p* = 0.008), and higher perceived health status (U = 40.0, *p* = 0.008), compared with the control group. Objective post-intervention measures of handgrip strength and chair-rise performance did not differ significantly between groups. Correlation analysis revealed strong associations between SARC-F score, muscle strength, functional mobility, and perceived health. Conclusions: The combined intervention consisting of a polyphenol-rich diet that influence gut microbiota and SarcoDYN^®^ supplementation, delivered alongside a structured rehabilitation program, was associated with better patient-reported mobility and health perception in adults at risk of sarcopenia. These findings should be considered exploratory and hypothesis-generating, and require confirmation in larger controlled studies.

## 1. Introduction

Sarcopenia is a progressive, age-related disorder of skeletal muscle characterized by a decline in muscle mass and physical performance. It is driven by an imbalance between protein synthesis and degradation, as well as mitochondrial dysfunction, chronic low-grade inflammation, and neuromuscular alterations. These pathophysiological changes lead to reduced muscle strength, impaired functional capacity, and increased vulnerability to falls, disability, hospitalization, and mortality. Although originally described as a geriatric condition, sarcopenia is now recognized as a broader clinical entity that may occur earlier in adulthood in the presence of risk factors such as physical inactivity, inadequate protein intake, vitamin D deficiency, multimorbidity, or prolonged immobilization. According to the EWGSOP2 consensus, reduced muscle strength represents the primary indicator of sarcopenia, while muscle quantity and quality are used to confirm the diagnosis, and physical performance reflects severity. Early identification and targeted interventions—including resistance training, optimized protein intake, and specific dietary and nutritional supplementation strategies—are essential to prevent progression and improve functional outcomes. In this context, combined approaches that address both nutritional and functional deficits have gained increasing attention as promising strategies to support muscle health in individuals at risk of sarcopenia.

There is currently an increasing body of evidence highlighting the importance of the dietary quality, bioactive food components, and targeted supplementation with specific micronutrients [1]. These factors may influence key biological pathways involved in age-related muscle decline, including inflammatory processes, oxidative stress, and metabolic regulation, and may also interact with the gut microbiota, thereby contributing to the development and progression of sarcopenia.

Polyphenol-rich diets have been shown to exert beneficial effects, including anti-inflammatory and antioxidant properties, improved mitochondrial function, and a positive influence on the balance between anabolic and catabolic processes. These effects are essential for maintaining muscle mass and function in older adults [2] and may also contribute to increased social participation and a greater sense of social inclusion, which are important at any age [3,4,5,6]. Available evidence suggests that a diet rich in polyphenols may play an important role in preserving muscle health and reducing frailty in ageing populations.

In particular, studies on the Mediterranean diet indicate that adherence to its principles is associated with better physical performance, increased muscle strength, and a lower risk of age-related frailty. These benefits are likely related to the intake of polyphenols, omega-3 fatty acids, and other bioactive compounds found in fruits, vegetables, legumes, whole grains, and extra virgin olive oil [7,8]. Flavonoids, phenolic acids, and stilbenes exhibit anti-inflammatory, antioxidant, and mitochondrial-modulating properties, which may mitigate the effects of oxidative stress, inflammation, and impaired energy metabolism in ageing muscle tissue [9,10].

Several studies have demonstrated that specific polyphenolic compounds, such as resveratrol, quercetin, and catechins, can influence inflammatory signaling pathways (e.g., NF-κB), oxidative stress responses (e.g., Nrf2 activation), and mitochondrial biogenesis (via PGC-1α), thereby potentially attenuating the processes that contribute to sarcopenia [11,12,13].

Studies in animal models consistently demonstrate that several polyphenolic compounds can attenuate muscle atrophy by modulating catabolic and mitochondrial pathways [9,10]. Resveratrol, a polyphenolic stilbene found in grapes and other plants, has emerged as a potential strategy for reducing muscle atrophy in preclinical models, particularly when used alongside other interventions. Its pleiotropic actions include modulation of the ubiquitin–proteasome system (UPS), enhancement of mitochondrial biogenesis via PGC-1α-related pathways, and reduction in oxidative stress and inflammation.

The effects of resveratrol have been investigated in various animal models representing different atrophy scenarios, including denervation, disuse/unloading, and sarcopenic obesity. In these contexts, resveratrol administration has been associated with attenuation of muscle atrophy and preservation of muscle structure [14,15]. Using a denervation-induced atrophy mouse model, Asami et al. (2018) demonstrated that a diet containing 0.5% resveratrol significantly reduced the loss of gastrocnemius muscle mass and fiber atrophy, which was associated with decreased immunoreactivity for atrogin-1 and p62 in muscle fibers [16].

In a study by Bennett et al. (2013) [17] using an aged rat model of hindlimb suspension (disuse), resveratrol did not prevent muscle mass loss during the unloading phase but significantly improved recovery of muscle mass and cross-sectional area of type IIA/IIB fibers during the subsequent rehabilitation period. These effects were associated with increased AMPK and PGC-1α signaling, suggesting that resveratrol may preferentially facilitate post-atrophy regeneration rather than providing full protection during acute disuse.

The interaction between obesity and sarcopenia has also been investigated using high-fat diet (HFD) models. In a mouse model combining HFD and physical inactivity, Huang et al. (2019) [18] showed that resveratrol attenuated features of sarcopenic obesity by improving mitochondrial function and suppressing activation of the ubiquitin–proteasome pathway in skeletal muscle. Mechanistically, resveratrol reversed mitochondrial dysfunction and oxidative stress, while attenuating activation of the ubiquitin–proteasome system, effects partly mediated by PKA/LKB1/AMPK signaling.

Taken together, these findings suggest that resveratrol may represent a promising adjunctive strategy for mitigating muscle atrophy and sarcopenia through dual mechanisms: reducing protein degradation (via the ubiquitin–proteasome system and autophagy) and enhancing mitochondrial and oxidative capacity (involving SIRT1, AMPK, and PGC-1α pathways).

Epigallocatechin-3-gallate (EGCG), the major bioactive polyphenol in green tea, has demonstrated multifaceted protective effects against sarcopenia in animal models, acting through antioxidant, anti-catabolic, and mitochondrial-preserving mechanisms. Oxidative stress, a key contributor to sarcopenia, leads to mitochondrial dysfunction, activation of catabolic pathways, and muscle fiber atrophy. EGCG exhibits strong antioxidant properties by directly scavenging reactive oxygen species and enhancing endogenous antioxidant defenses. Studies in ageing rodents models have shown that EGCG reduces lipid peroxidation, protein carbonylation, and mitochondrial oxidative injury [19,20]. Due to its accumulation within mitochondria, EGCG provides targeted protection of mitochondrial DNA and respiratory chain complexes from oxidative damage [20,21]. Meador et al. demonstrated in aged rats that EGCG suppresses key proteolytic mediators of muscle degradation, including the E3 ligases MuRF1 and MAFbx, which are involved in the ubiquitin–proteasome pathway [20]. In addition, EGCG downregulates myostatin and proteasome subunits, while upregulating anabolic factors such as IGF-1 and IL-15. Furthermore, EGCG has been shown to support mitochondrial integrity by increasing the expression of key regulators of mitochondrial biogenesis, including PGC-1α, NRF1, and TFAM, while reducing mitochondrial apoptotic signaling. These effects contribute to improved mitochondrial function and cellular energetics in ageing muscle [20]. Additional evidence indicates that EGCG enhances mitochondrial oxidative capacity and metabolic efficiency in aged skeletal muscle. Collectively, these findings suggest that EGCG modulates central molecular pathways involved in age-related muscle degeneration, reducing oxidative damage, inhibiting proteolytic signaling, and improving mitochondrial function. As a result, EGCG attenuates sarcopenic muscle loss in preclinical models. However, further research is needed to confirm these effects in humans, and well-designed clinical trials are required to evaluate the therapeutic potential of EGCG.

Curcumin is a natural polyphenolic compound widely investigated for its anti-inflammatory and antioxidant properties. It has been shown to modulate multiple molecular targets, including transcription factors, cytokines, enzymes, and signaling pathways involved in oxidative stress and inflammation. Curcumin has been extensively studied in animal models of muscle atrophy, demonstrating its ability to suppress nuclear factor-κappa B (NF-κB) activation and attenuate catabolic processes in skeletal muscle [22]. In a rat model of sepsis, Poylin et al. (2008) demonstrated that curcumin significantly reduces NF-κB activation in skeletal muscle, leading to decreased expression of components of the ubiquitin-proteasome pathway and attenuation of sepsis-induced muscle proteolysis [23]. Similarly, in streptozotocin-induced type 1 diabetic mice, curcumin supplementation was shown to downregulate inflammatory cytokines and NF-κB activity, suppress muscle-specific E3 ligases, and preserve muscle mass and fiber size [24]. In aged mice exposed to chronic exercise stress, curcumin reduced sarcopenia-related changes by attenuating oxidative stress, lowering inflammatory signaling, and improving the balance between muscle protein synthesis and degradation [25]. Furthermore, studies in rat models of cachexia have demonstrated that curcumin mitigates muscle wasting by inhibiting NF-κB-mediated transcriptional activity and reducing proteasome function [26]. Collectively, these findings support the role of curcumin as a key modulator of NF-κB signaling, capable of attenuating muscle atrophy across various pathological conditions.

While these preclinical findings suggest a therapeutic potential for mitigating sarcopenia, studies conducted in human populations have yielded more limited and heterogeneous results [27]. Clinical investigations of resveratrol in older adults have generally demonstrated improvements in mitochondrial enzyme activity and oxidative capacity, but have not consistently shown increases in muscle mass or strength [28,29]. Similarly, studies evaluating EGCG and curcumin in humans have highlighted their anti-inflammatory and metabolic benefits; however, their direct effects on muscle hypertrophy and functional performance remain modest [30,31]. These effects may be partially comparable to rehabilitation strategies that primarily target inflammation and joint mobility rather than inducing substantial anabolic adaptations [32,33,34,35].

Overall, current human data suggest that the benefits of polyphenols are relatively moderate, underscoring the need for larger, well-designed clinical trials. Importantly, phenolic compounds may interact with key anabolic nutrients such as leucine, vitamin D, and magnesium at multiple levels, including gut microbiota modulation, intestinal absorption, and intracellular signaling pathways, thereby potentially enhancing their biological effects [36,37].

The relationship between polyphenols and the gut microbiota is bidirectional. Intestinal bacteria metabolize complex phenolic compounds into lower-molecular-weight metabolites with improved bioavailability and biological activity, facilitating systemic absorption and downstream metabolic actions. Conversely, polyphenols can selectively promote the growth of beneficial bacterial taxa and modulate microbial diversity, contributing to improved gut barrier function and reduced inflammation. Through these reciprocal mechanisms, microbiota-mediated transformations may influence anabolic signaling, micronutrient bioavailability, and cellular metabolic responses, potentially amplifying the combined effects of leucine, vitamin D, and magnesium within a multimodal nutritional strategy.

The management of sarcopenia is widely recognized to depend strongly on adequate nutritional support, particularly protein supplementation [38]. Among essential amino acids, leucine has gained considerable attention due to its unique ability to stimulate muscle protein synthesis through specific cellular signaling mechanisms. Leucine acts both as a substrate for protein synthesis and as a metabolic signal, activating the mammalian target of rapamycin complex 1 (mTORC1) pathway, which regulates translation initiation and promotes muscle anabolism [39,40].

Through this signaling role, leucine enhances the efficiency of muscle protein synthesis and supports anabolic processes critical for muscle maintenance and growth. Several studies have demonstrated that older adults require higher leucine intakes (~2.5–3 g per meal) to achieve muscle protein synthesis responses comparable to those of younger individuals [41,42]. The intake of leucine-enriched protein sources therefore appears effective in counteracting this blunted anabolic response.

Beyond leucine, vitamin D3 also plays a key role in muscle physiology. Adequate vitamin D status contributes to improved muscle fiber function, calcium homeostasis, and neuromuscular performance, while deficiency has been consistently associated with reduced strength, impaired balance, and increased risk of falls in older adults. Vitamin D3 supplementation has been shown to support muscle performance, particularly in individuals with insufficient baseline levels [43], and may also contribute to functional improvements following structured rehabilitation programs [44].

Magnesium is another key micronutrient required for optimal muscle function, as it plays a central role in ATP production, muscle contraction and relaxation, and serves as a cofactor for enzymes involved in protein synthesis. Low magnesium levels have been associated with reduced muscle performance, increased inflammation, and impaired mitochondrial function in older adults. Conversely, adequate magnesium intake has been linked to improved muscle strength and physical performance, supporting its relevance as part of a multimodal approach to sarcopenia management [45,46,47].

In this context, integrating targeted nutritional strategies with structured rehabilitation may represent a promising approach to address both the metabolic and functional components of sarcopenia. While individual interventions such as protein supplementation, vitamin D optimization, or polyphenol-rich diets have shown partial benefits, evidence regarding their combined effects—particularly in relation to gut microbiota modulation and patient-centered functional outcomes—remains limited.

Therefore, the aim of the present study was to evaluate whether a multimodal intervention consisting of a polyphenol-rich dietary recommendation combined with leucine, vitamin D3, and magnesium supplementation (SarcoDYN^®^), integrated into a standardized rehabilitation program, provides superior improvements in functional and patient-reported outcomes compared with rehabilitation alone in adults at risk of sarcopenia.

This quasi-experimental, non-randomized retrospective study focused on patient-relevant outcomes, with the SARC-F score at follow-up defined as the primary endpoint due to its clinical utility as a screening and functional assessment tool. Secondary outcomes included muscle strength, sit-to-stand performance, perceived mobility, and perceived health status, allowing a comprehensive evaluation of both objective and subjective dimensions of functional recovery.

## 2. Materials and Methods

### 2.1. Study Design and Setting

This quasi-experimental, non-randomized retrospective controlled study was conducted over a 3-month period within the Research Core of the Physical and Rehabilitation Medicine Department (RMFB Section). The objective was to evaluate the effects of combining a nutritional supplement containing leucine, vitamin D3, and magnesium with a diet rich in natural phenolic compounds on muscle strength and performance in adult patients diagnosed with sarcopenia.

### 2.2. Participants and Eligibility Criteria

The study population consisted of adult patients aged 18–89 years who were diagnosed with sarcopenia according to the European Working Group on Sarcopenia in Older People (EWGSOP2) algorithm.

The inclusion criteria were:

- Age between 18 and 89 years;
- Confirmed diagnosis of sarcopenia based on the EWGSOP2 criteria;
- Compliance with the prescribed supplementation, dietary recommendations, and individualized physiotherapy program.

Exclusion criteria were:

- Age below 18 years or above 89 years;
- Incomplete data at any of the required evaluation points;
- Presence of severe comorbidities that prevent assessment of physical performance, completion of the rehabilitation program, or adherence to the treatment protocol.
- Known hypervitaminosis D or contraindications to vitamin D supplementation.

All patients who met the predefined eligibility criteria during the study period and had complete data at both evaluation time points were included in the final analysis.

### 2.3. Diagnostic Algorithm for Sarcopenia

The diagnostic process followed the European Working Group on Sarcopenia in Older People (EWGSOP2) recommendations.

Initially, all participants completed the SARC-F questionnaire as a screening tool for sarcopenia suspicion. Subsequently, the following assessments were performed:

- Handgrip strength test, measured using a handheld dynamometer for both hands. According to EWGSOP2, low muscle strength was defined as <27 kg for men and <16 kg for women.
- Chair stand test, recording the time required to rise five times from a seated position without using the arms. A time ≥ 15 s indicated reduced lower limb strength.
- Physical performance assessment, performed using either:
  • Gait speed test (<0.8 m/s indicated low performance), or;
  • Timed Up and Go (TUG) test (≥20 s indicated low performance).

According to the operational definition:

- Probable sarcopenia was defined by low muscle strength only;
- Confirmed sarcopenia by low strength and reduced muscle quantity/quality;
- Severe sarcopenia by low strength, reduced muscle quantity/quality, and poor physical performance.

Although the diagnostic framework followed EWGSOP2 recommendations, the application of inclusion and exclusion criteria was pragmatic and adapted to real-world clinical conditions, integrating both diagnostic and feasibility considerations.

### 2.4. Intervention and Control Protocols

Participants were classified as having probable sarcopenia according to EWGSOP2 criteria, based on reduced muscle strength and functional performance, in the absence of direct assessments of muscle quantity or quality (e.g., DXA, BIA, or ultrasound), which were not available in this setting.

Participants were retrospectively selected from a cohort of 360 patients evaluated during the first six months of 2025. The selection process involved sequential exclusion of individuals with abnormal vitamin D levels, neurocognitive disorders affecting motor or cognitive performance, lack of adherence to the rehabilitation program, dietary recommendations, or supplementation protocol, as well as those who did not meet the predefined criteria for reduced muscle strength and functional impairment.

After applying all eligibility criteria, a total of 28 participants were included in the final analysis. A detailed participant flow diagram illustrating recruitment, exclusions, and group allocation is presented in the Section 3 (Results).

Eligible participants were assigned to one of two groups based on a pragmatic, real-world allocation process. Group assignment was determined through shared decision-making between the patient and the clinician, with mutual agreement on the selected treatment approach, taking into account patient preference, availability and adherence to oral supplementation (SarcoDYN^®^, Actafarma, Madrid, Spain), access to recommended foods within the polyphenol-rich diet, and compliance with the prescribed home-based kinesiotherapy program.

Study group (Group 1):

Participants received oral nutritional supplementation consisting of leucine, vitamin D3, and magnesium, administered as SarcoDYN^®^, one sachet dissolved in a glass of water daily, for a continuous period of three months. Each sachet contained:

- β-hydroxy-β-methylbutyrate (HMB)—3 g (a metabolite of leucine);
- Vitamin D3—7.5 μg (300 IU; 150% NRV);
- Magnesium—300 mg (80% NRV).

In addition, all participants followed a polyphenol-rich diet, emphasizing the daily intake of fresh fruits and vegetables (such as grapes, grapefruit, oranges, apples, tomatoes, and broccoli), as well as olives, whole cereals, nuts, tea, and cocoa. The polyphenol-rich dietary intervention included the recommendation that patients consume at least one item from the designated list at each meal. Adherence to this approach was high, as participants expressed a preference for improving their health through natural dietary sources rather than pharmacological treatments or dietary supplements. This preference was likely influenced by the fact that many patients were already undergoing multiple medical therapies for their comorbidities. The supplementation in the study group was provided on top of these dietary recommendations and the structured rehabilitation program followed by all participants.

All participants in this group also underwent a supervised physiotherapy program aimed at improving exercise capacity and endurance, restoring activities of daily living, and increasing global muscle tone.

The physiotherapy regimen included isotonic and isometric strengthening exercises (with or without weights) that can help to increase the muscular strength like other physical modalities [48,49,50] and aerobic training to enhance cardiovascular fitness and resistance to sustained effort [51,52,53].

Patients initially completed a two-week supervised rehabilitation program within a specialized department. They associated during hospitalization other exercises for their chronic musculoskeletal disorders, including osteoarthritis, discopathies, enthesopathies, tendinopathies, and osteoporosis. Upon discharge, they received structured recommendations for a comprehensive exercise regimen targeting both upper and lower limbs, aimed at improving muscle strength and aerobic endurance. This program included the use of light weights, pedaling devices, and activities such as walking or treadmill exercise. At the discharge, the rehabilitation program was prescribed at a minimum frequency of four sessions per week, with a duration of at least 30 min per day performed at home. The type of exercises, along with their intensity and progression, were individualized and established during hospitalization.

Control group (Group 2):

Patients followed the same physiotherapy program for three months but did not receive nutritional supplementation or dietary recommendations for polyphenol intake.

### 2.5. Evaluation and Outcome Measures

Participants were evaluated at two time points:

- T0—baseline (study inclusion);
- T1—after 3 months (post-intervention).

The following parameters were analyzed:

- Muscle strength (handgrip and lower limb performance);
- Functional performance;
- Patient-reported outcomes (SARC-F score, perceived mobility, perceived health status);
- Correlations among functional, strength, and subjective variables.

### 2.6. Statistical Analysis

Statistical analyses were performed using IBM SPSS Statistics version 27.0 (IBM Corp., Armonk, NY, USA) and Microsoft Excel 2024 (Microsoft Corp., Redmond, WA, USA). Data distribution was assessed using the Shapiro–Wilk test and visual inspection of histograms and Q–Q plots [54]. Continuous variables with normal distribution were analyzed using parametric tests, whereas skewed or ordinal variables were evaluated using non-parametric methods.

Between-group comparisons at baseline and post-intervention were performed using the independent samples *t*-test for normally distributed variables, and the Mann–Whitney U test for non-normally distributed or ordinal outcomes [55]. Categorical or ordinal variables with multiple levels and small expected cell counts were analyzed using the Fisher–Freeman–Halton Exact test with Monte Carlo estimation [56].

Within-group pre–post changes were analyzed using the paired samples *t*-test for normally distributed variables and the Wilcoxon signed-rank test for non-parametric or ordinal data [57]. Effect sizes for parametric analyses were reported using Cohen’s d, and rank-biserial correlation coefficients were computed for non-parametric tests [58].

Correlations between functional performance, strength, and subjective outcomes were assessed using Spearman’s rank correlation coefficient (rho) due to the non-normality and ordinal nature of several variables. All tests were two-tailed, and a *p*-value < 0.05 was considered statistically significant.

The primary outcome of the study was defined as the SARC-F score at follow-up (T2), given its clinical relevance as a screening and functional assessment tool in individuals at risk of sarcopenia. All other outcomes, including muscle strength, sit-to-stand performance, perceived mobility, and perceived health status, were considered secondary or exploratory.

Given the relatively small sample size and the exploratory nature of several endpoints, no formal adjustment for multiple comparisons was applied. Instead, effect sizes and the overall consistency of findings across outcomes were taken into consideration when interpreting the results.

In addition to post-intervention comparisons, change-score (Δ) analyses were performed to explore differences in improvement between groups. These analyses were considered exploratory and interpreted with caution, particularly in the context of a non-randomized design.

Although an analysis of covariance (ANCOVA) approach adjusting for baseline values is often recommended in such settings, this method was not applied due to the small sample size and the non-normal distribution of several variables. Therefore, non-parametric methods were retained as the primary analytical approach.

### 2.7. Ethical Considerations

The study was conducted in accordance with the principles of the Declaration of Helsinki. All participants provided written informed consent prior to inclusion. The study protocol was approved by the Ethics Committee of Colentina Clinical Hospital (approval no. 15/17 June 2025). All patient data were anonymized, and confidentiality was strictly maintained throughout the research process.

## 3. Results

**Figure 1:**
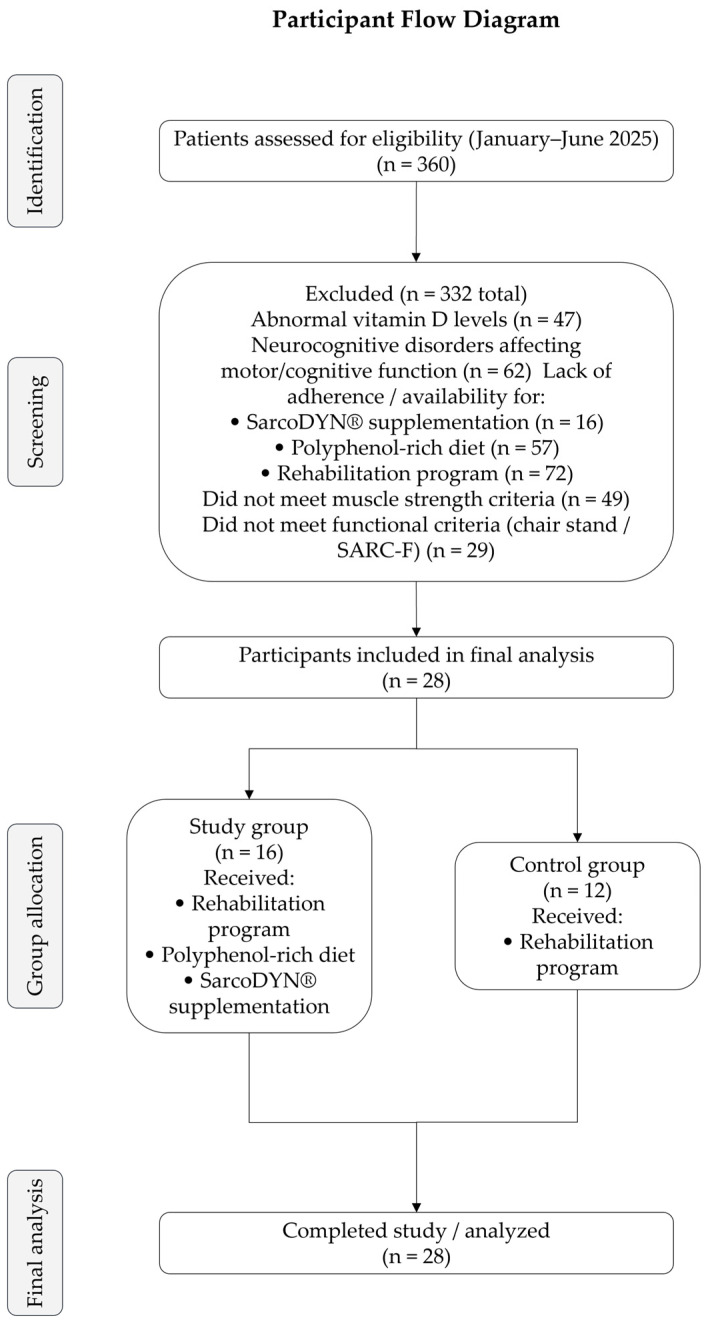
Participant flow diagram.

A total of 360 patients were retrospectively assessed for eligibility between January and June 2025. After applying the predefined inclusion and exclusion criteria, 28 participants met the eligibility criteria and were included in the final analysis. The participant selection process is presented in Figure 1.

Among these, 16 participants were assigned to the study group (rehabilitation + polyphenol-rich diet + SarcoDYN^®^ supplementation), while 12 were included in the control group (rehabilitation only). Group allocation followed a pragmatic approach based on adherence, availability, and shared decision-making between the patient and the clinician.

Flowchart illustrating the selection process of participants, including initial screening, exclusion criteria, and final allocation into study and control groups. Participants were retrospectively selected from patients evaluated between January and June 2025. Group allocation was based on adherence, availability, and shared decision-making between patient and clinician.

### 3.1. Baseline Characteristics of the Study Groups (Table 1)

At baseline, no statistically significant differences were identified between the control group (*n* = 12) and the study group (*n* = 16) with respect to age, BMI, initial SARC-F score, dominant and non-dominant handgrip strength, functional mobility (up-from-chair time), perceived mobility, or perceived health status (all *p* > 0.05; Table 1). These findings indicate that the two groups were comparable prior to the intervention, supporting the interpretation that any post-intervention differences are unlikely to be explained by baseline disparities.

**Table 1:** Baseline characteristics of the study population.

Variable	Control Group (*n* = 12)	Study Group (*n* = 16)	Statistical Test	*p*-Value
Age (yrs.)	69.50 [66.50–75.00]	71.50 [67.25–78.75]	Mann–Whitney U	0.530
BMI (kg/m^2^)	28.10 [24.86–33.91]	29.90 [27.90–33.45]	Mann–Whitney U	0.546
SARC-F score (initial)	5.50 [5.00–7.75]	4.50 [4.00–7.00]	Mann–Whitney U	0.196
Dominant Muscle Strength (initial)	15.00 [11.00–18.75]	13.00 [11.00–22.00]	Mann–Whitney U	0.944
Non-Dominant Muscle Strength (initial)	12.13 ± 4.55	13.84 ± 6.62	Independent *t*-test	0.218
Up from Chair (s)	21.00 [17.25–41.75]	27.50 [20.00–38.00]	Mann–Whitney U	0.560
Mobility grade (initial)	4.00 [3.25–5.75]	5.00 [4.00–7.00]	Mann–Whitney U	0.212
Health status (initial)	4.50 [4.00–6.75]	5.00 [4.25–7.00]	Mann–Whitney U	0.367

Note: Data are presented as median [IQR] for non-normally distributed variables and as mean ± standard deviation (SD) for normally distributed variables. Between-group comparisons were performed using the Mann–Whitney U test or independent samples *t*-test, as appropriate.

Normality of continuous variables was evaluated using the Shapiro–Wilk test. Because most baseline measures showed non-normal distribution in at least one group and the sample size was small, baseline continuous and ordinal outcomes were summarized as median [IQR] and compared between groups using the Mann–Whitney U test. Non-dominant handgrip strength was additionally checked using a parametric comparison, yielding consistent results.

Overall, participants in both groups started the rehabilitation program from similar functional, muscular, and perceptual levels.

### 3.2. Within-Group Changes in Functional, Strength, and Perceptual Parameters (Table 2)

Within-group changes from baseline (T0) to follow-up (T1) were analyzed separately for the control group (CG, *n* = 12) and the study group (SG, *n* = 16). Depending on the distribution of each variable, paired-samples *t*-tests were applied for normally distributed continuous outcomes, while Wilcoxon Signed-Rank tests were used for non-normally distributed or ordinal variables. Effect sizes were reported as Cohen’s d for paired *t*-tests and as r for Wilcoxon analyses. For parametric comparisons, mean differences with 95% confidence intervals were also considered to support interpretation of effect size.

**Table 2:** Within-Group Changes in Functional and Strength Parameters.

Variable	Statistical Test	Pre Mean ± SD	Post Mean ± SD	Statistic	*p*-Value	Effect Size
SARC-F score						
Control Group	Wilcoxon	6.17 ± 1.85	5.50 ± 2.24	Z = −2.530	0.011	r = 0.730
Study Group	Wilcoxon	5.38 ± 1.59	2.88 ± 1.59	Z = −3.580	<0.001	r = 0.895
Dominant Muscle Strength						
Control Group	Paired *t*-test	15.25 ± 4.31	15.83 ± 4.35	t(11) = −2.028	0.067	d = 0.586
Study Group	Wilcoxon	15.69 ± 6.89	19.41 ± 8.62	Z = −3.433	0.001	r = 0.858
Non-Dominant Muscle Strength						
Control Group	Paired *t*-test	12.13 ± 4.55	12.50 ± 4.89	t(11) = −1.092	0.298	d = 0.315
Study Group	Paired *t*-test	13.84 ± 6.62	16.66 ± 7.86	t(15) = −5.306	<0.001	d = 1.326
Up from Chair						
Control Group	Wilcoxon	29.17 ± 15.00	27.17 ± 15.34	Z = −1.616	0.106	r = 0.466
Study Group	Wilcoxon	31.31 ± 16.20	21.88 ± 12.61	Z = −3.524	<0.001	r = 0.881
Perceived Mobility Score						
Control Group	Wilcoxon	4.50 ± 1.57	5.00 ± 1.95	Z = −2.121	0.034	r = 0.612
Study Group	Wilcoxon	5.25 ± 1.44	7.00 ± 1.16	Z = −3.337	0.001	r = 0.834
Perceived Health Status Score						
Control Group	Wilcoxon	5.08 ± 1.44	5.58 ± 1.83	Z = −2.121	0.034	r = 0.612
Study Group	Wilcoxon	5.56 ± 1.50	7.50 ± 1.16	Z = −3.572	0.001	r = 0.893

Note: Values are reported as mean ± SD. Within-group comparisons were performed using paired-samples *t*-tests for normally distributed variables and Wilcoxon Signed-Rank tests for non-normally distributed or ordinal variables. Effect sizes are expressed as Cohen’s d for paired *t*-tests and as r for Wilcoxon tests (r = |Z|/√N).

#### 3.2.1. SARC-F Score

SARC-F score decreased significantly in both groups from baseline to follow-up, indicating reduced self-reported functional limitations.

In the control group, the improvement was statistically significant (Z = −2.530, *p* = 0.011; effect size r = 0.73).

Similarly, the study group demonstrated a significant reduction in SARC-F score (Z = −3.580, *p* < 0.001; effect size r = 0.90), reflecting a large within-group effect.

#### 3.2.2. Muscle Strength

In the control group, dominant handgrip strength showed a non-significant increase from 15.25 ± 4.31 kg to 15.83 ± 4.35 kg (t(11) = −2.028, *p* = 0.067; effect size Cohen’s d = 0.586; 95% CI: −0.05 to 1.22). Non-dominant handgrip strength increased slightly from 12.13 ± 4.55 kg to 12.50 ± 4.89 kg, without statistical significance (t(11) = −1.092, *p* = 0.298; effect size Cohen’s d = 0.315;95% CI: −0.38 to 1.13).

In the study group, dominant handgrip strength improved significantly from 15.69 ± 6.89 kg to 19.41 ± 8.62 kg (Wilcoxon Z = −3.433, *p* = 0.001; effect size Cohen’s d = 0.858). Similarly, non-dominant handgrip strength increased from 13.84 ± 6.62 kg to 16.66 ± 7.86 kg (t(15) = −5.306, *p* < 0.001; effect size Cohen’s d = 1.326; 95% CI: 1.68 to 3.94).

#### 3.2.3. Functional Mobility (Up-from-Chair Time)

In the control group, the time required to rise from a chair decreased from 29.17 ± 15.00 s to 27.17 ± 15.34 s, but this change was not statistically significant (Wilcoxon Z = −1.616, *p* = 0.106; effect size Cohen’s d = 0.466).

In contrast, the study group demonstrated a significant reduction in up-from-chair time, from 31.31 ± 16.20 s at baseline to 21.88 ± 12.61 s at follow-up (Wilcoxon Z = −3.524, *p* < 0.001; effect size Cohen’s d = 0.881).

#### 3.2.4. Perceived Mobility and Perceived Health Status

Perceived mobility improved in both groups. In the control group, scores increased from 4.50 ± 1.57 to 5.00 ± 1.95 (Wilcoxon Z = −2.121, *p* = 0.034; effect size = 0.612). In the study group, perceived mobility improved from 5.25 ± 1.44 to 7.00 ± 1.16 (Wilcoxon Z = −3.337, *p* = 0.001; effect size Cohen’s d = 0.834).

A similar pattern was observed for perceived health status. The control group showed an improvement from 5.08 ± 1.44 to 5.58 ± 1.83 (Wilcoxon Z = −2.121, *p* = 0.034; effect size Cohen’s d = 0.612), while the study group increased from 5.56 ± 1.50 to 7.50 ± 1.16 (Wilcoxon Z = −3.572, *p* = 0.001; effect size Cohen’s d = 0.893).

Overall, both groups exhibited improvements across multiple outcomes; however, changes were more pronounced and consistently statistically significant in the study group, particularly for functional mobility and patient-reported measures.

### 3.3. Between-Group Differences in Post-Intervention Outcomes (Table 3)

Between-group comparisons were performed at follow-up (T1) to evaluate whether participants receiving the combined nutritional intervention (SarcoDYN^®^ supplementation and a polyphenol-rich diet) in addition to the standardized rehabilitation program achieved superior outcomes compared with those undergoing rehabilitation alone. Given the non-normal distribution of most variables, Mann–Whitney U tests were used for ordinal or non-normally distributed outcomes, while independent-samples *t*-tests were applied for normally distributed continuous variables. Effect sizes were calculated as r = |Z|/√N for Mann–Whitney tests and as Cohen’s d for the independent *t*-test.

**Table 3:** Between-group post-intervention comparisons (follow-up values).

Variable	Statistical Test	Post CG Mean ± SD	Post SG Mean ± SD	Statistic	*p*-Value	Effect Size
SARC-F final	Mann–Whitney	5.50 ± 2.24	2.88 ± 1.59	U = 30.0; Z = −3.101	0.002	r = 0.586
Dominant Muscle Strength (final)	Mann–Whitney	15.83 ± 4.35	19.41 ± 8.62	U = 81.5; Z = −0.677	0.498	r = 0.128
Non-Dominant Muscle Strength (final)	Independent *t*-test	12.50 ± 4.89	16.66 ± 7.86	t(26) = −1.608	0.120	d = 0.614
Up from Chair (final)	Mann–Whitney	27.17 ± 15.34	21.88 ± 12.61	U = 77.0; Z = −0.886	0.376	r = 0.167
Mobility grade (final)	Mann–Whitney	5.00 ± 1.95	7.00 ± 1.16	U = 40.0; Z = −2.657	0.008	r = 0.5
Health status (final)	Mann–Whitney	5.58 ± 1.83	7.50 ± 1.16	U = 40.0; Z = −2.648	0.008	r = 0.5

#### 3.3.1. SARC-F Score (Follow-Up)

At follow-up, the study group reported significantly lower SARC-F scores compared with the control group, indicating fewer functional limitations. The Mann–Whitney U test confirmed a statistically significant difference between groups (U = 30.0, Z = −3.101, *p* = 0.002), with a large effect size (r = 0.59). Mean ranks were higher in the control group (20.00) than in the study group (10.38), supporting a better functional profile among supplemented participants.

#### 3.3.2. Dominant Muscle Strength (Follow-Up)

Dominant handgrip strength at follow-up did not differ significantly between groups. The Mann–Whitney analysis showed no statistically significant group effect (U = 81.5, Z = −0.677, *p* = 0.498), indicating comparable dominant-side strength outcomes after the intervention period (small effect size, r = 0.13).

#### 3.3.3. Non-Dominant Muscle Strength (Follow-Up)

Non-dominant muscle strength was higher in the study group compared with the control group at follow-up; however, the independent-samples *t*-test did not confirm statistical significance (t(26) = −1.608, *p* = 0.120; 95% CI: −9.47 to 1.16). The effect size was moderate (Cohen’s d = 0.61), suggesting a potentially meaningful trend that did not reach statistical significance in the present sample.

#### 3.3.4. Functional Mobility (Up-from-Chair Time, Follow-Up)

The functional mobility outcome (up-from-chair time) did not show significant differences between groups at follow-up. Mann–Whitney testing indicated no statistically significant group effect (U = 77.0, Z = −0.886, *p* = 0.376), with a small effect size (r = 0.17), showing that both groups achieved comparable objective functional performance.

#### 3.3.5. Perceived Mobility (Follow-Up)

In contrast, perceived mobility scores were significantly higher in the study group compared with the control group at follow-up, reflecting greater self-reported improvement. Mann–Whitney analysis showed a statistically significant between-group difference (U = 40.0, Z = −2.657, *p* = 0.008), with a large effect size (r = 0.50).

#### 3.3.6. Perceived Health Status (Follow-Up)

A similar pattern was observed for perceived global health status, which was significantly better in the study group. The Mann–Whitney U test confirmed a statistically significant difference (U = 40.0, Z = −2.648, *p* = 0.008), also associated with a large effect size (r = 0.50).

Overall, between-group comparisons at follow-up revealed that participants receiving the combined nutritional intervention (SarcoDYN^®^ supplementation and a polyphenol-rich diet) achieved significantly better patient-reported outcomes, including lower SARC-F scores and higher perceived mobility and health status. In contrast, between-group differences were not significant for dominant muscle strength and objective functional mobility, while non-dominant strength showed a moderate but non-significant trend favoring the study group.

### 3.4. Between-Group Differences in Changes Scores (Table 4)

To further evaluate whether the study group achieved greater improvements compared with the control group, between-group comparisons were performed on change scores (Δ = follow-up − baseline) using Mann–Whitney U tests.

**Table 4:** Between-group comparison of change scores (Δ = follow-up − baseline).

Variable	U	Z	*p*-Value	Effect Size
Δ SARC-F global score	4.50	−4.415	<0.001	r = 0.834
Δ Dominant Muscle Strength	14.50	−3.846	<0.001	r = 0.727
Δ Non-Dominant Muscle Strength	15.00	−3.820	<0.001	r = 0.722
Δ Up from Chair	27.50	−3.20	0.001	r = 0.605
Δ Mobility grade	35.50	−2.954	0.003	r = 0.558
Δ Health status	21.00	−3.707	<0.001	r = 0.701

#### 3.4.1. Δ SARC-F Global Score

The reduction in SARC-F global score was significantly greater in the study group compared with the control group (U = 4.5, Z = −4.415, *p* < 0.001), indicating a markedly stronger improvement in global functional status among participants receiving the combined nutritional intervention (SarcoDYN^®^ supplementation and a polyphenol-rich diet). The effect size was large (r = 0.835).

#### 3.4.2. Δ Dominant Muscle Strength

The study group showed significantly greater gains in dominant handgrip strength compared with the control group (U = 14.5, Z = −3.846, *p* < 0.001), with a large effect size (r = 0.727).

#### 3.4.3. Δ Non-Dominant Muscle Strength

Similarly, improvement in non-dominant handgrip strength was significantly greater in the study group (U = 15.0, Z = −3.820, *p* < 0.001), also demonstrating a large effect size (r = 0.722).

#### 3.4.4. Δ Up-from-Chair Performance

Change in up-from-chair time differed significantly between groups (U = 27.5, Z = −3.200, *p* = 0.001). The direction of change favored the study group, reflecting a more pronounced functional improvement. The effect size was large (r = 0.605).

#### 3.4.5. Δ Perceived Mobility

Perceived mobility improved significantly more in the study group than in the control group (U = 35.5, Z = −2.954, *p* = 0.003), with a large effect size (r = 0.558).

#### 3.4.6. Δ Perceived Health Status

Likewise, perceived health status showed significantly greater improvement in the study group (U = 21.0, Z = −3.707, *p* < 0.001), with a large effect size (r = 0.701).

Overall, the study group demonstrated consistently greater gains across all functional, strength, and patient-reported outcomes when comparing change scores, supporting an added benefit of the combined nutritional strategy (SarcoDYN^®^ supplementation and a polyphenol-rich diet) when integrated into the rehabilitation and dietary program.

### 3.5. Correlation Analysis

Spearman’s rank correlation analyses were conducted to investigate associations between baseline functional status, post-intervention outcomes, and improvement scores across the entire cohort (*n* = 28). The correlation matrix included SARC-F total scores (baseline and follow-up), sarcopenia risk classification (follow-up), handgrip strength (dominant and non-dominant), up-from-chair performance, perceived mobility and perceived health status, as well as change scores (Δ) for key outcomes.

#### 3.5.1. Baseline and Follow-Up SARC-F Scores

Baseline SARC-F total score was strongly correlated with follow-up SARC-F total score (ρ = 0.849, *p* < 0.001), indicating good internal consistency of functional limitation severity across time. Baseline SARC-F was also positively associated with follow-up sarcopenia risk (ρ = 0.730, *p* < 0.001), suggesting that higher perceived functional burden at baseline was linked to a higher probability of remaining at risk at follow-up.

In addition, baseline SARC-F correlated negatively with both baseline strength measures (dominant: ρ = −0.616, *p* < 0.001; non-dominant: ρ = −0.684, *p* < 0.001), as well as with baseline perceived mobility (ρ = −0.716, *p* < 0.001) and perceived health status (ρ = −0.652, *p* < 0.001). These associations confirm that worse initial functional perception aligned with lower muscular capacity and poorer self-reported health-related outcomes.

#### 3.5.2. Follow-Up SARC-F Score and Global Recovery Profile

At follow-up, higher SARC-F total score remained strongly associated with poorer recovery-related outcomes. Specifically, follow-up SARC-F correlated negatively with dominant (ρ = −0.599, *p* < 0.001) and non-dominant strength (ρ = −0.711, *p* < 0.001), as well as with perceived mobility (ρ = −0.762, *p* < 0.001) and perceived health status (ρ = −0.809, *p* < 0.001).

Moreover, follow-up SARC-F correlated positively with follow-up up-from-chair time (ρ = 0.606, *p* < 0.001), indicating that participants with higher residual functional limitations also demonstrated slower sit-to-stand performance.

#### 3.5.3. Sarcopenia Risk at Follow-Up

Follow-up sarcopenia risk classification showed significant negative correlations with post-intervention muscle strength (dominant: ρ = −0.454, *p* = 0.015; non-dominant: ρ = −0.571, *p* = 0.002), and with perceived mobility and health status (mobility grade: ρ = −0.701, *p* < 0.001; health status grade: ρ = −0.648, *p* < 0.001). These results indicate that participants who remained classified at risk tended to exhibit reduced muscle capacity and poorer subjective recovery perception.

#### 3.5.4. Relationships Between Objective Performance and Perceived Outcomes

Baseline and follow-up up-from-chair times were strongly correlated (ρ = 0.835, *p* < 0.001), suggesting stable performance ranking across participants. Follow-up up-from-chair time showed significant inverse associations with perceived mobility (ρ = −0.509, *p* = 0.006) and perceived health status (ρ = −0.561, *p* = 0.002), supporting the relationship between slower functional performance and poorer subjective appraisal.

Perceived mobility and perceived health status were strongly interrelated both at baseline (ρ = 0.810, *p* < 0.001) and at follow-up (ρ = 0.848, *p* < 0.001), indicating coherent perception of recovery in terms of mobility and general health.

#### 3.5.5. Correlations Involving Improvement Scores (Δ Outcomes)

Change-score analyses demonstrated that improvement in muscle strength was closely aligned between limbs. Dominant and non-dominant strength gain were strongly correlated (ρ = 0.817, *p* < 0.001). Strength improvement was also associated with improvements in perceived recovery: dominant strength gain correlated positively with mobility improvement (ρ = 0.460, *p* = 0.014) and health status improvement (ρ = 0.561, *p* = 0.002), while non-dominant strength gain correlated with mobility improvement (ρ = 0.611, *p* < 0.001) and health improvement (ρ = 0.661, *p* < 0.001).

Functional improvement in the up-from-chair test (Δ time reduction) was significantly associated with better perceived recovery: up-from-chair improvement correlated inversely with mobility improvement (ρ = −0.560, *p* = 0.002) and health improvement (ρ = −0.538, *p* = 0.003). This indicates that larger functional gains were accompanied by stronger subjective improvement.

Finally, SARC-F improvement (Δ score reduction) was strongly linked with perceived improvements in both mobility (ρ = −0.666, *p* < 0.001) and health status (ρ = −0.724, *p* < 0.001), highlighting that reduction in perceived functional limitation was accompanied by meaningful perceived recovery benefits.

**Figure 2:**
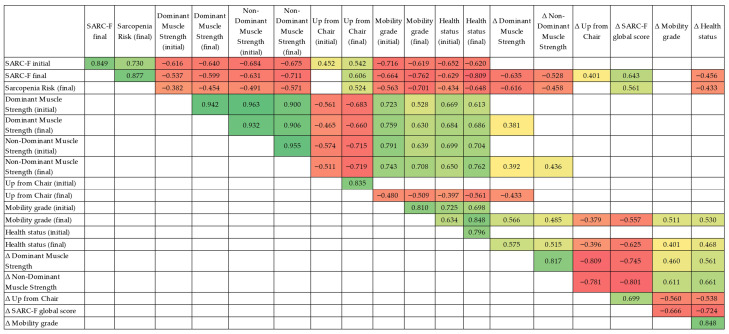
Correlation heatmap. The heatmap uses a green-to-red color scale to represent Spearman correlation coefficients, where green indicates positive correlations, red indicates negative correlations, and color intensity reflects the strength of the association (range: −1 to +1). Only statistically significant correlations are shown.

Correlation patterns are illustrated in the heatmap (Figure 2), highlighting strong associations between SARC-F burden, perceived mobility/health, muscle strength, and chair-rise performance.

The results of change-score analyses were consistent with the post-intervention comparisons; however, these findings should be interpreted with caution given the non-randomized design and the potential influence of baseline variability.

Data on SarcoDYN^®^ treatment tolerability were collected and indicated an overall very good profile. Only a small number of patients, more accurate two patients reported difficulties at administration due to an unpleasant taste without any other adverse reactions or incompatibilities.

## 4. Discussion

The present quasi-experimental non-randomized controlled study examined the effects of a structured rehabilitation program on functional performance, muscle strength, and patient-reported outcomes in adults at risk of sarcopenia. Outcomes were compared between a standard intervention (control group) and an enhanced protocol (study group) that included oral supplementation with a leucine-derived formulation (SarcoDYN^®^) combined with a polyphenol-rich diet, alongside the same exercise-based rehabilitation program. Overall, both groups demonstrated clinically relevant improvements over time; however, the study group achieved superior outcomes at follow-up in several key patient-reported domains, suggesting an added value of the enhanced intervention particularly for perceived mobility and general health status.

### 4.1. Within-Group Improvements

Within-group analyses revealed improvements across multiple domains in both study arms, although the magnitude and statistical consistency differed between groups.

In the study group, significant improvements were observed for the majority of parameters, including reductions in SARC-F total score and up-from-chair time, alongside improvements in perceived mobility and perceived health status. Notably, muscle strength increased significantly in both dominant and non-dominant hands, with large effect sizes for the non-dominant limb. These findings support the responsiveness of functional mobility, subjective perceptions, and strength markers to structured multimodal rehabilitation, particularly when combined with nutritional supplementation.

In contrast, the control group showed a more limited pattern of change. While significant improvements were identified in self-reported mobility and health status and in SARC-F total score, objective strength indicators did not demonstrate statistically significant within-group improvement, and the up-from-chair test also did not change significantly. This discrepancy may indicate that while standard rehabilitation can improve self-perceived functional capacity and health-related impressions, measurable strength gains may require additional biological support or higher training intensity and volume than provided by the base protocol.

Importantly, these results suggest that patient-reported outcomes may be more sensitive to early functional changes than purely performance-based measures, especially in participants with probable sarcopenia and heterogeneous baseline functional profiles. Also, the anti-inflammatory effect of polyphenolic components expressed on joints mobility could contribute at the better outcomes for self-reported mobility and health status and in SARC-F total score. This observation is consistent with previous findings showing that higher-quality dietary patterns, such as Mediterranean-style adherence, are associated with improvements in fatigue, functional capacity, and activities of daily living in older or clinical populations, supporting the relevance of diet-related factors for patient-reported outcomes [59].

### 4.2. Between-Group Differences

Although both groups improved clinically, several follow-up outcomes clearly differentiated the study group from the control group. Most notably, the final SARC-F score was significantly lower in the study group, indicating fewer perceived functional limitations after the intervention. Similarly, both perceived mobility and perceived health status were significantly higher in the study group at follow-up, reflecting a superior subjective response to the enhanced protocol.

In contrast, objective performance outcomes such as muscle strength and chair-rise time did not consistently differ significantly between groups when assessed only at follow-up. This pattern suggests that the core exercise-based program contributed to general improvement across the entire cohort, while the enhanced intervention primarily strengthened patient-reported outcomes and perceived functional autonomy. From a clinical perspective, this is highly relevant, as subjective mobility and health perception strongly influence long-term adherence, self-efficacy, and willingness to engage in continued activity.

Overall, the direction and magnitude of significant between-group differences consistently favored the enhanced intervention, particularly for SARC-F, perceived mobility, and perceived health status outcomes, potentially reflecting stronger engagement, higher perceived benefit, or greater responsiveness to the combined rehabilitation and supplementation strategy.

### 4.3. Correlational Structure of Functional, Strength, and Perceptual Variables

Correlation analyses provided an integrated framework for interpreting the relationships between functional performance, strength, and patient-reported outcomes. Both baseline and follow-up SARC-F scores showed strong associations with multiple outcome measures, confirming SARC-F as a robust indicator of functional vulnerability and perceived limitation in adults at risk of sarcopenia.

Final SARC-F scores demonstrated meaningful negative correlations with dominant and non-dominant handgrip strength and with mobility and health status grades, and positive correlations with up-from-chair time. This pattern supports the multidimensional nature of sarcopenia-related impairment, where reduced strength, reduced functional efficiency, and poorer self-rated health tend to cluster in the same individuals.

Additionally, the inclusion of improvement indices (Δ scores) contributed clinically useful information. Improvement in global SARC-F score correlated with gains in mobility and health perception, emphasizing that subjective recovery is not an isolated outcome, but rather part of a coherent functional trajectory involving both physical and perceptual dimensions.

### 4.4. Clinical Relevance

From a rehabilitation perspective, the combination of clinically meaningful within-group improvements and statistically significant between-group advantages in patient-reported outcomes supports the practical value of enhanced protocols for individuals at risk of sarcopenia. Even when objective gains are modest or inconsistent, improvements in perceived mobility and perceived health status may represent important therapeutic achievements, as these dimensions are closely linked to independence, confidence during movement, and long-term behavioral adherence.

### 4.5. Interpretation in the Context of Sarcopenia Risk

The observed associations between functional outcomes, strength markers, and SARC-F evolution support the concept that sarcopenia risk is not purely defined by muscle strength, but by a combined profile of functional performance and perceived limitations. This perspective is further supported by the well-recognized dilemma between weight loss and muscle preservation in older adults, where improvements in metabolic health may coexist with a risk of muscle mass decline if nutritional strategies are not appropriately balanced [60]. The superior subjective outcomes observed in the study group suggest that integrating specific diet–microbiota interactions and nutritional supplementation into early rehabilitation may enhance the perceived effectiveness of intervention and may support functional independence, particularly in individuals with early-stage decline or probable sarcopenia where diagnosis cannot be fully confirmed using advanced imaging methods.

### 4.6. Strengths and Limitations

This study benefits from the combined evaluation of objective and subjective outcomes, allowing a multidimensional understanding of rehabilitation response in adults at risk of sarcopenia. The inclusion of both within-group testing and between-group comparisons supports a comprehensive interpretation of change over time.

However, several limitations should be acknowledged. The absence of randomization may have introduced unmeasured baseline differences (e.g., motivational factors, lifestyle variability, or behavioral adherence), which may partially explain the stronger subjective response observed in the study group. In addition, the lack of direct muscle mass quantification (e.g., DXA or BIA-derived indices) limits diagnostic confirmation and restricts interpretation to probable sarcopenia or risk status. The relatively short follow-up period further limits conclusions regarding the durability of the observed improvements.

From a statistical perspective, the analysis was constrained by the relatively small sample size and the absence of a prespecified multiplicity adjustment, which may increase the risk of type I error for secondary outcomes. In addition, the limited sample size reduces statistical power and increases the risk of type II error, potentially leading to underestimation of true effects.

Furthermore, although change-score analyses were used to explore between-group differences in improvement, such approaches may be influenced by regression to the mean or subtle baseline imbalances in non-randomized designs. Future studies should consider randomized allocation and more robust analytical approaches, such as ANCOVA or mixed-effects models, to better account for baseline variability.

### 4.7. Implications for Rehabilitation Practice

The results highlight the importance of integrating patient-reported outcomes into routine monitoring of sarcopenia-oriented rehabilitation. In this context, plant-forward dietary strategies rich in bioactive compounds, including polyphenols, are increasingly recognized for their potential to modulate inflammation, gut microbiota, and metabolic pathways, thereby supporting functional outcomes in clinical populations [61]. While objective strength and performance measures remain essential, subjective mobility and health perception provide complementary insight into how individuals experience functional recovery [62,63,64]. Enhanced protocols combining structured rehabilitation with targeted diet strategies and supplementation may offer added value in improving perceived independence and overall functional confidence, which are critical determinants of long-term outcomes.

In summary, the present findings indicate that a structured rehabilitation program can generate meaningful functional and patient-reported benefits in adults at risk of sarcopenia. Importantly, the enhanced protocol combining a polyphenol-rich diet and SarcoDYN^®^ supplementation was associated with greater improvements in subjective mobility, perceived health status, and SARC-F outcomes, supporting the potential value of integrating nutritional strategies into rehabilitation interventions in this population. The present findings should be interpreted as exploratory and hypothesis-generating, providing a basis for future randomized controlled studies.

## 5. Conclusions

This quasi-experimental study showed that a structured rehabilitation program was associated with meaningful improvements in functional capacity and patient-reported outcomes in adults at risk of sarcopenia. Within-group analyses indicated significant reductions in SARC-F total score and improvements in perceived mobility and perceived health status in both groups, while objective strength and chair-rise performance improved more consistently in the study group receiving diet recommendations and supplementation.

Importantly, participants who received a polyphenol-rich diet in addition to SarcoDYN^®^ supplementation and standardized rehabilitation achieved significantly better follow-up outcomes in key subjective domains, including lower final SARC-F scores and higher self-rated mobility and health status, compared with the control group. Although between-group differences were less evident for objective post-intervention strength and chair-rise time, the direction of change and the overall pattern of improvement supported a favorable clinical profile for the enhanced protocol.

Correlation findings further confirmed strong relationships between functional limitation (SARC-F), muscle strength, mobility performance, and perceived recovery, emphasizing the multidimensional character of sarcopenia-related vulnerability.

Overall, these results suggest that integrating a combined nutritional strategy (a polyphenol-rich diet that interact with gut microbiota and SarcoDYN^®^ supplementation) into a rehabilitation-based approach may provide additional benefits, particularly for patient-reported functional recovery. Larger controlled studies with extended follow-up and objective confirmation of muscle mass/quality are warranted to clarify the long-term clinical relevance of this combined strategy.

## Data Availability

The data presented in this study are available on request from the corresponding authors. The data are not publicly available due to privacy and ethical restrictions.
